# Kartagener syndrome with pulmonary mycetoma and recurrent thromboembolism: A case report

**DOI:** 10.1097/MD.0000000000051057

**Published:** 2026-10-09

**Authors:** Muhammad Asghar Khan, Okasha Tahir, Abbas Khan, Maheen Sheraz, Khalid Reza, Muhammad Hassaan Javaid, Laleen Altaf, Eeman Shehzad, Muhammad Ali, Hasibullah Aminpoor, Tayyeb Ali

**Affiliations:** a Department of cardiology, Hayatabad Medical Complex HMC, Peshawar, Pakistan; b Department of Medicine, Khyber medical university, Peshawar, Pakistan; c Department of Medicine, Continental Medical College, Lahore, Pakistan; d Department of Medicine, Enam Medical College, Dhaka, Bangladesh; e Department of Medicine, Shifa College of Medicine, Islamabad, Pakistan; f Faculty of Medicine, Kabul University of Medical Sciences “Abu Ali Ibn Sina,” Kabul, Afghanistan; g Gomal Medical College, Dera Ismail Khan, Pakistan.

**Keywords:** Bronchiectasis, Deep vein thrombosis, Kartagener syndrome, Primary ciliary dyskinesia, Pulmonary mycetoma, Situs inversus totalis

## Abstract

**Rationale:**

Kartagener syndrome, a variant of primary ciliary dyskinesia, is defined by chronic sinusitis, bronchiectasis, and situs inversus. While recurrent infections are seen, a combination of thromboembolic disease, pulmonary mycetoma, cardiac dysfunction, and serological evidence of autoimmune disorders is very uncommon.

**Patient concerns:**

A 27-year-old man with Kartagener syndrome and situs inversus totalis complaining of worsening dyspnea, chest pain, fever, hemoptysis, and ankle swelling. His past medical history included episodes of deep vein thrombosis, recurrent pulmonary embolism, and chronic productive cough.

**Diagnoses:**

Radiologic studies showed dextrocardia, a right upper lobe cavitary lesion indicative of pulmonary mycetoma, hepatic congestion, and ascites. The echocardiogram demonstrated severe dysfunction of both left and right ventricles, a thrombus in the right ventricle, mild-moderate pericardial effusion, and an ejection fraction of about 40%. BNP was elevated, and U1-ribonucleoprotein antibodies were positive, implying a probable overlap of autoimmune disease, but diagnostic criteria were not fulfilled.

**Interventions:**

He was prescribed appropriate heart failure management, anticoagulant therapy, antifungals, and multidisciplinary treatment of other issues.

**Outcomes and Lessons:**

The patient underwent adequate management of his cardiopulmonary, infectious, and thromboembolic complications. The existence of a causal link between these diseases could not be found. This case underscores the need for a multidisciplinary approach in evaluating patients with a combination of congenital and acquired systemic diseases. The association of Kartagener syndrome, pulmonary mycetoma, thromboembolic tendency, right ventricular thrombus formation, cardiac dysfunction, and positive U1-RNP is an unusual combination and calls for proper evaluation.

## 1. Introduction

Kartagener syndrome is a rare autosomal recessive disorder and a subtype of primary ciliary dyskinesia (PCD), characterized by the classical triad of situs inversus, chronic sinusitis, and bronchiectasis. The underlying defect in ciliary motility predisposes affected individuals to recurrent respiratory infections and progressive structural lung disease.^[1]^ Situs inversus totalis is typically asymptomatic, but may complicate the clinical course by altering anatomical orientation and occasionally delaying diagnosis.^[2]^

The association of superimposed pulmonary mycetoma in patients suffering from Kartagener syndrome is uncommon. It was described by Nakagawa and Shimazu in a case of Kartagener syndrome where a patient developed a pulmonary aspergilloma associated with previous tuberculosis that was treated effectively with antifungal drugs.^[3]^In this report, the term “pulmonary mycetoma” refers to an Aspergillus-associated intracavitary fungal ball (aspergilloma) developing within a preexisting pulmonary cavity. Similar findings were also found in a study conducted by Ashitani and coworkers where aspergilloma complicated bronchiectatic cavities in 2 cases of Kartagener syndrome who suffered from hemoptysis.^[4]^ All these cases support of the theory that structural defects produced by chronic ciliary dysfunction and bronchiectasis make way for fungal infection; however, such combinations are less common.In contrast, recurrent venous thromboembolism with right ventricular thrombus has rarely been reported in the context of Kartagener syndrome. To our knowledge, the combination of recurrent thromboembolic disease, right ventricular thrombus, and pulmonary mycetoma in a patient with Kartagener syndrome has not been well characterized in the available literature.

In addition, the presence of recurrent venous thromboembolism, right ventricular thrombus, biventricular dysfunction, and pericardial effusion associated with U1-RNP antibodies implies a potential overlap with connective tissue disease.^[5,6]^ The coexistence of congenital ciliary disease, Aspergillus-associated pulmonary mycetoma, recurrent thromboembolism, cardiac dysfunction, and U1-RNP-positive serology represents an unusual clinical constellation. To our knowledge, this combination has rarely been described in a single patient.

## 2. Case presentation

A structured timeline of the patient’s clinical course is summarized in Table 1.

**Table 1:** Timeline of clinical events and findings in the patient.

Time point	Clinical event/finding
Several years before current admission	Thrombophlebitis complicated by deep vein thrombosis (DVT); managed with laser surgery. Kartagener syndrome with situs inversus totalis diagnosed during the same evaluation.
Intermediate period	Chronic dry cough developed, progressing over time to a productive cough.
Within the past year	Productive cough progressed to hemoptysis. Pulmonary embolism (PE) diagnosed on CT imaging with elevated D-dimer. Abdominal ultrasound showed IVC and hepatic vein dilatation with ascites and hepatic congestion (Fig. 1).
Intermediate Period	MRI thorax showed dextrocardia (Fig. 4). CT confirmed dextrocardia with situs inversus totalis.
Months preceding current admission	Echocardiography: dextrocardia, globally hypokinetic left ventricle, mobile right ventricular apical thrombus with McConnell’s sign, severe biventricular dysfunction, mild-to-moderate pericardial effusion, ejection fraction ~40%, mild mitral/tricuspid regurgitation. Pro-BNP > 6000 pg/mL. U1-RNP antibodies positive (5.52%). HRCT chest: right upper lobe cavitary lesion consistent with pulmonary mycetoma with bilateral fibrotic changes (Figs. 2–3).
Current admission	Worsening exertional dyspnea, chest pain radiating to the jaw, fever, productive cough with hemoptysis, and pedal edema. SpO_2_ 88% on room air. Managed with anticoagulation, itraconazole, guideline-directed heart failure therapy, and supportive care.

A 27-year-old male with a history of Kartagener’s syndrome and situs inversus totalis developed symptoms of worsening exertional dyspnea, chest pain radiating to the jaw, fever, productive cough with streaky hemoptysis, and pedal edema. The diagnosis of Kartagener’s syndrome with situs inversus totalis had been made some years ago after an episode of thrombophlebitis associated with deep vein thrombosis (DVT) that had necessitated laser surgery. Advanced methods of diagnosing primary ciliary dyskinesia such as nasal nitric oxide measurement, ciliary biopsy with transmission electron microscopy, or DNA sequencing were not available at the institution where he was diagnosed; his diagnosis was based on clinical findings (recurring sinopulmonary infections) together with radiological demonstration of bronchiectasis and total situs inversus. The patient later developed a chronic dry cough that turned into a productive cough, with hemoptysis in the last year. A diagnosis of pulmonary embolism (PE) had been made about a year before this admission through contrast-enhanced CT of the chest and increased D-dimer (>2.0 mg/L). He had since been on oral warfarin.

Information on family history related to consanguinity, primary ciliary dyskinesia, premature venous thromboembolism, or connective tissue disease was not available for verification. Occupational history was not recorded; hence, the ability to assess any occupational exposure to fungi predisposing the patient to pulmonary mycetoma was limited. The patient did not have a history of any drug allergies. The patient’s previous treatment before the current admission involved a combination of treatments given after he was diagnosed with PE, resulting in right ventricular strain:

Furosemide 40 mg per day (diuretics for fluid overload), Finerenone 10 mg per day (non-steroidal mineralocorticoid receptor agonist), Dapagliflozin 10 mg per day (SGLT2 inhibitors in heart failure management), Digoxin 0.25 mg per day (inotropic agents), Vericiguat 2.5 mg per day (soluble guanylate cyclase stimulators in chronic heart failure management), Warfarin 5 mg per day (long-term anticoagulant in PE). Smoking and alcohol consumption details were not given in the available information.

### 2.1. Investigations

Ultrasonographic examination done almost one year prior to the current admission had shown IVC and hepatic vein dilatation with ascites and hepatic congestion (Fig. 1). The thoracic MRI study had revealed dextrocardia (Fig. 4). The CT scan showed dextrocardia with situs inversus totalis. An echocardiogram done in the months preceding admission had shown dextrocardia with situs inversus totalis, global hypokinesis of the left ventricle, right ventricular apical thrombus with McConnell’s sign (consistent with PE), severe biventricular dysfunction, mild to moderate pericardial effusion, ejection fraction of about 40%, and mild mitral and tricuspid regurgitation. Pro-BNP was high (>6000 pg/mL). The autoantibody panel was positive for U1-RNP antibody (5.52%), raising the possibility of an underlying connective tissue disease process. However, the available clinical and serological data were insufficient to establish a diagnosis of mixed connective tissue disease. Further workup for other connective tissue disease panels (ANA, anti-dsDNA, anti-Sm, anti-Scl-70, anti-Ro/SSA, anti-La/SSB, rheumatoid factor) and clinical evidence for MCTD (Raynaud’s phenomenon, sclerodactyly, inflammatory arthritis) was not documented; therefore, classification criteria (Alarcón-Segovia or Kasukawa) were not fulfilled. Chest HRCT scan showed a cavitary lesion in the right upper lobe suggestive of pulmonary mycetoma with bilateral fibrotic changes predominantly on the right side (Figs. 2–3). Although microbiological confirmation was not available before admission, subsequent investigations supported an Aspergillus-associated pulmonary mycetoma. Serum Aspergillus IgG precipitins were positive, the serum galactomannan index was 1.6, and Aspergillus fumigatus was isolated from sputum culture. These findings, together with the characteristic cavitary lesion containing an intracavitary soft-tissue density on chest CT, supported the diagnosis.

**Figure 1.**
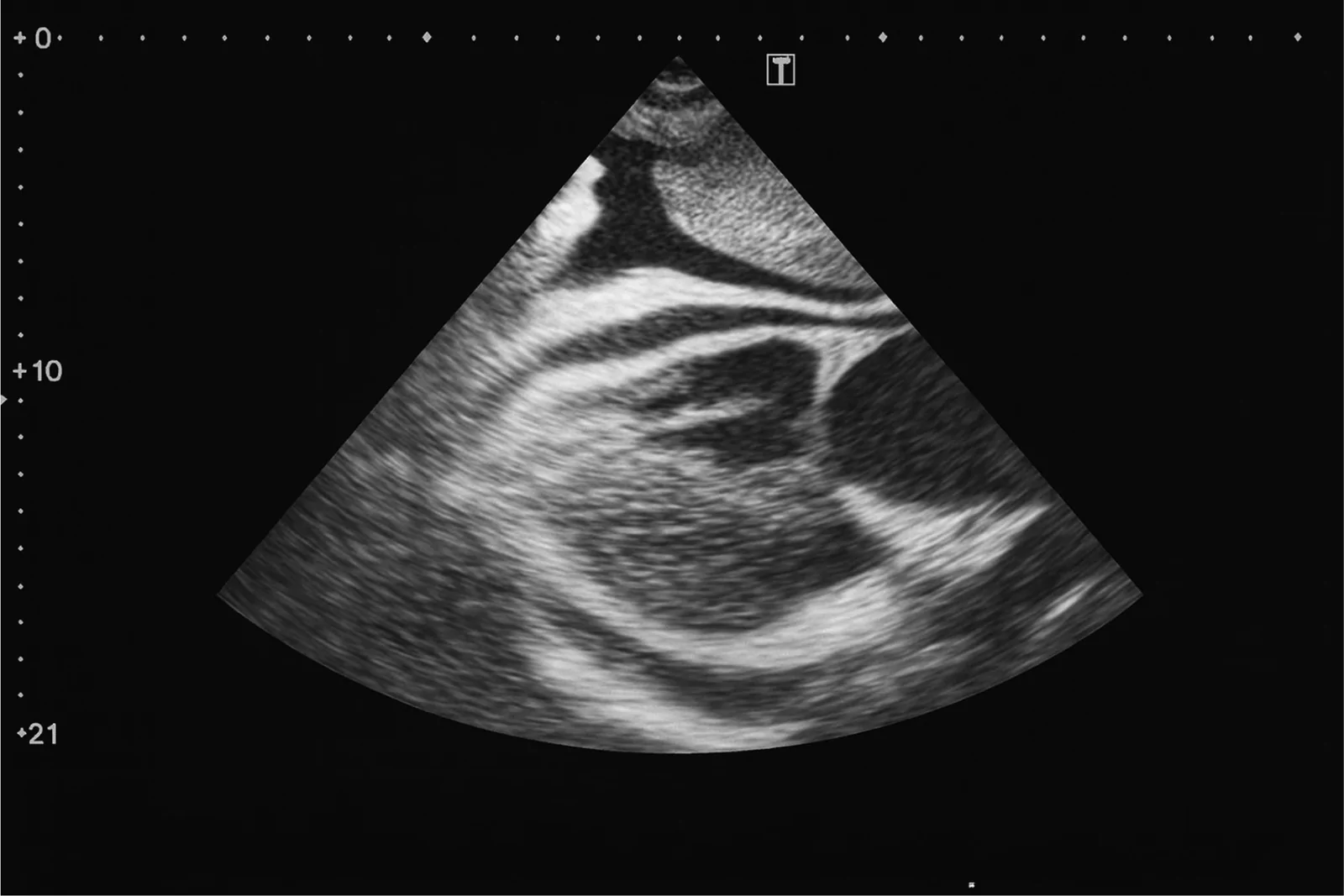
Abdominal ultrasound showing inferior vena cava (IVC) and hepatic vein dilatation with associated ascites and hepatic congestion, consistent with right heart strain/congestive hepatopathy.

**Figure 2.**
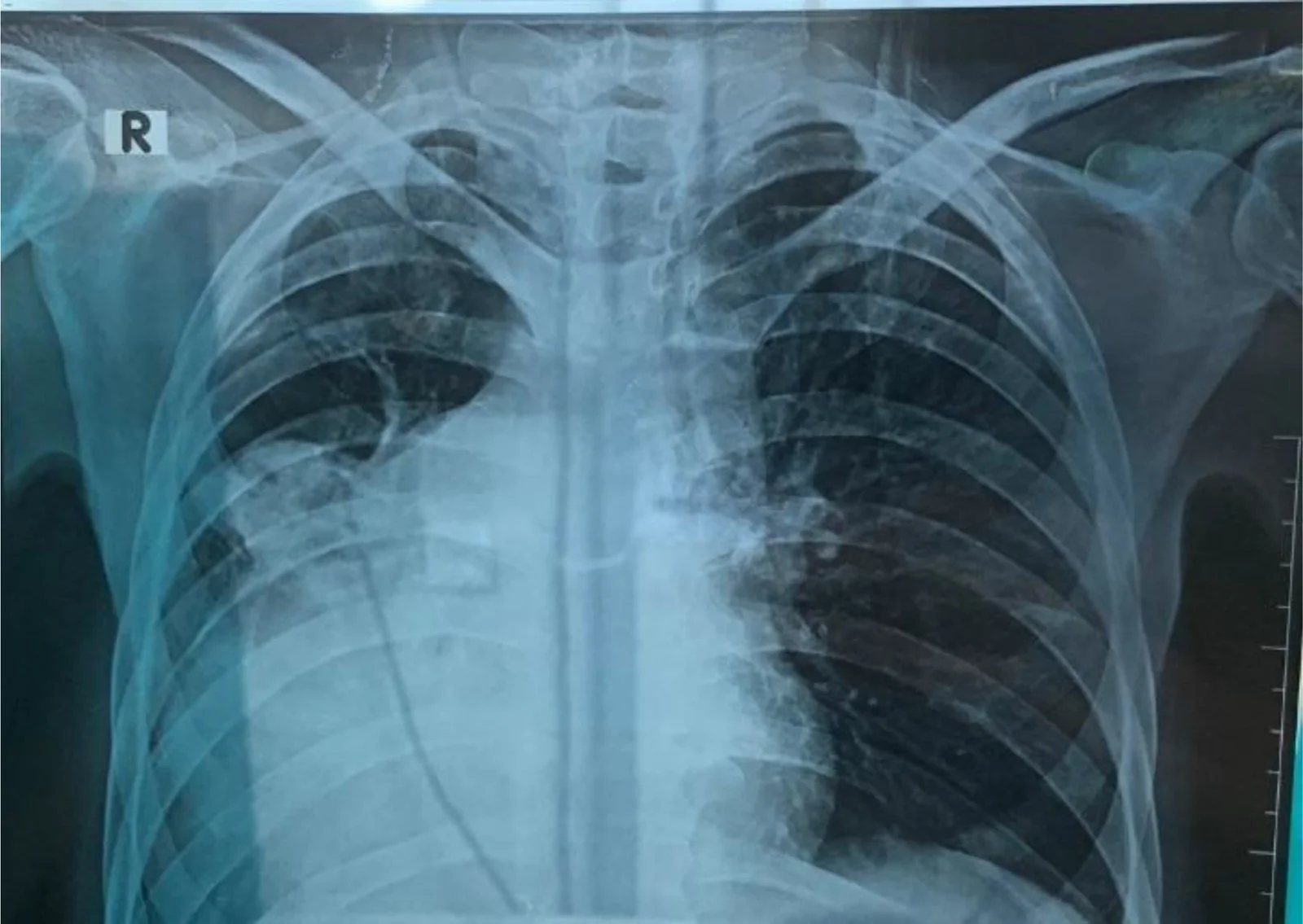
Chest radiograph demonstrating dextrocardia with a rightward-oriented cardiac apex and right-sided gastric bubble, confirming situs inversus totalis.

**Figure 3.**
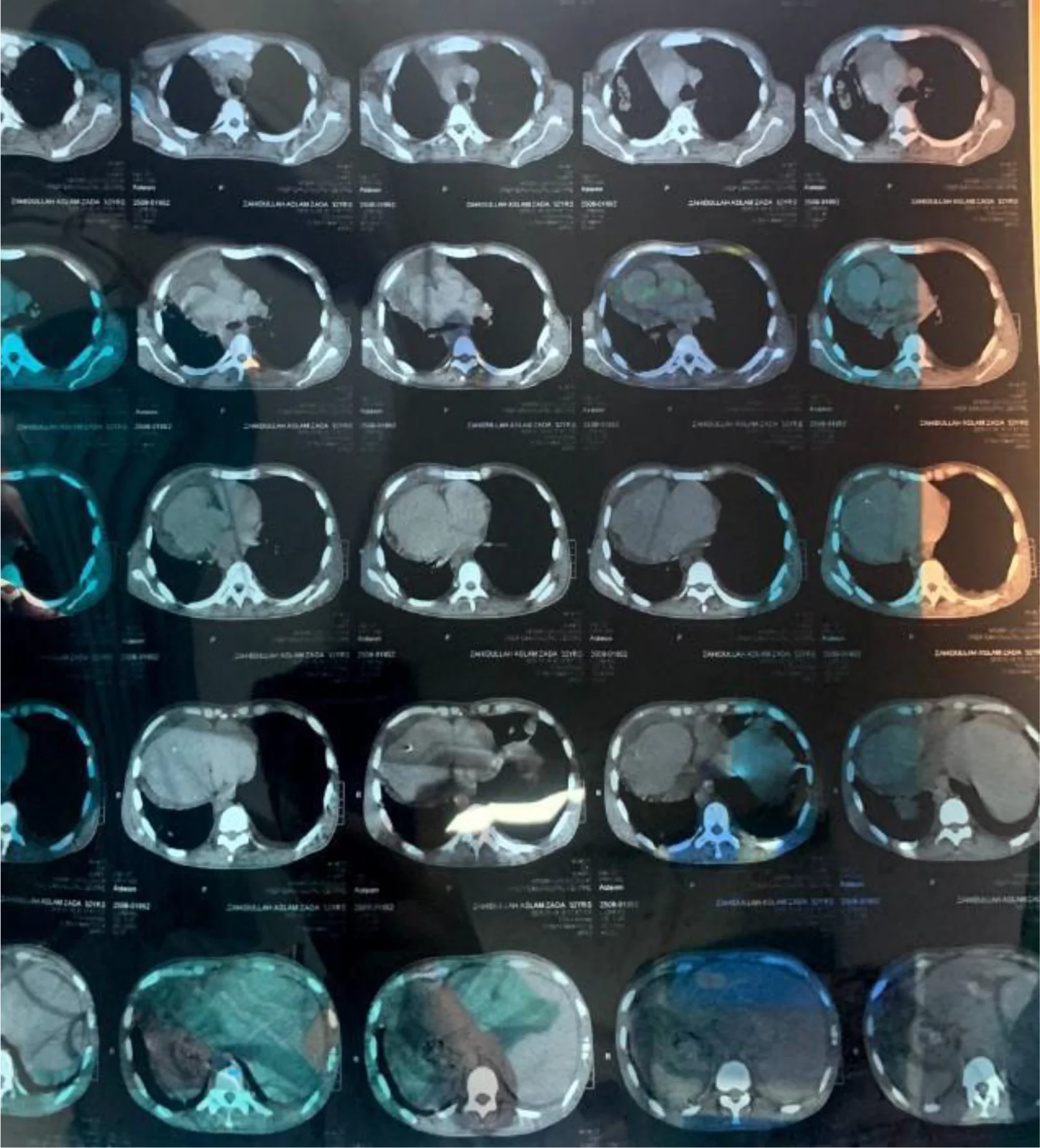
CT chest demonstrating a right upper lobe cavitary lesion containing an intracavitary soft-tissue density compatible with an Aspergillus-associated pulmonary mycetoma (aspergilloma), with associated bilateral bronchiectatic and fibrotic changes.

**Figure 4.**
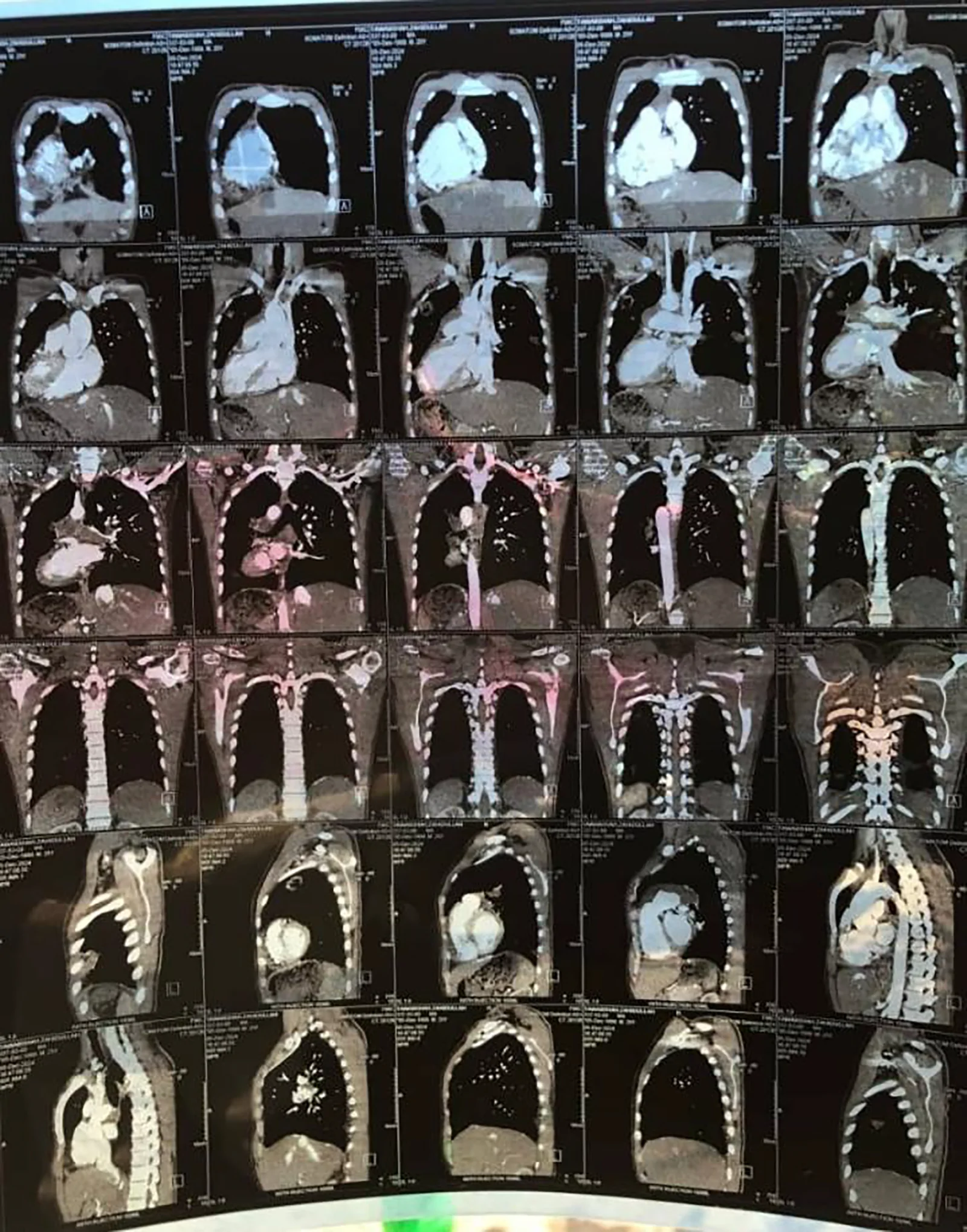
T1-weighted thoracic MRI confirming dextrocardia and situs inversus totalis.

On admission, BP was 100/80 mm Hg, HR was 78/min, O_2_ saturation was 88% in room air (improved to 97% on 10 L supplemental O_2_), and Glasgow Coma Scale was 15/15. Physical examination findings included decreased breath sounds bilaterally, audible S1 and S2 with murmurs due to mitral valve and tricuspid valve insufficiency, a soft, non-tender abdomen, pitting edema, and an unremarkable neurological examination. The laboratory investigations showed a total leukocyte count of 14.4 × 10^9^/L (neutrophils 74%, lymphocytes 15%), prothrombin time of 12 seconds, INR 1.0, and BNP 6126 pg/mL.Notably, the admission INR was 1.0 despite the reported use of warfarin 5 mg daily, indicating that anticoagulation was markedly subtherapeutic at presentation. This finding is clinically relevant given the patient’s history of recurrent DVT and pulmonary embolism and provides a plausible contributor to recurrent thromboembolic events. A comprehensive thrombophilia panel test was conducted during hospitalization. Protein C activity, protein S activity, antithrombin III levels, factor V Leiden mutation, and prothrombin G20210A gene mutation tests were within normal limits. Antiphospholipid antibodies test, which includes lupus anticoagulant, anticardiolipin antibodies (IgG/IgM), and anti-β_2-glycoprotein I antibodies (IgG/IgM) test results were also normal, indicating the absence of inherited or acquired classic thrombophilia.The recurrent thromboembolic events may have been related to a combination of subtherapeutic anticoagulation, chronic inflammation, and hypoxemia; however, a definitive causal relationship cannot be established from this case.

### 2.2. Management

During admission, management consisted of the use of intravenous analgesics/antipyretics (paracetamol under the brand name Provas), continuation of heart failure treatment according to guideline recommendations (finerenone 10 mg once daily, dapagliflozin 10 mg once daily, vericiguat 2.5 mg once daily, furosemide 40 mg once daily), and adjustment of anticoagulation.

For the pulmonary mycetoma, treatment with oral itraconazole 100 mg twice daily was started. Surgical treatment was not recommended due to the high risk for the patient, stemming from severe biventricular dysfunction, right ventricular thrombus, pulmonary hypertension/right ventricular strain, and cardiopulmonary instability as a whole. Thus, conservative management with antifungal treatment was performed along with clinical and radiological monitoring of the patient. Considering the possible interaction of itraconazole and warfarin, further anticoagulant therapy needed proper monitoring with the achievement of therapeutic INR values of 2.0 to 3.0 during the follow-up.

The patient had sustained clinical improvement after one, three, and 6 months of follow-up, with the cessation of dyspnea, hemoptysis, and peripheral edema. Therapy with warfarin was performed in such a way as to maintain INR values of 2.0 to 3.0. At 3 months, echocardiography revealed the resolution of right ventricular apical thrombus with the improvement of LVEF to 40% to 48% along with the regression of the pericardial effusion. Six months later, the HRCT revealed stable dimensions of the pulmonary cavity without an increase in the dimensions of intracavitary mycetoma and new parenchymal lesions.

## 3. Discussion

The above case provides an example of how all 4 types of conditions are observed in one patient diagnosed with Kartagener’s syndrome and situs inversus totalis. Although the temporal association of the mentioned conditions has clinical importance, the relationships of mechanisms described below remain hypothesis-generating due to the limitations of a case study.

Kartagener’s syndrome, a variant of Primary Ciliary Dyskinesia (PCD), features ciliary structural abnormalities resulting in reduced mucociliary clearance, recurrent infections, and bronchiectasis.^[1]^ In the case of our patient, the chronic nature of bronchiectasis likely served as a structural substrate for the growth of fungi, which is also seen in other patients suffering from pulmonary mycetoma in association with Kartagener’s syndrome.^[3,4]^ Mycetoma usually occurs in pre-formed cavities,^[7]^ and poor mucociliary clearance leads to infection.^[8]^ In this patient, radiological suspicion was confirmed on admission, based on high titers of Aspergillus IgG precipitins, a galactomannan index of 1.6, and culture of sputum showing Aspergillus fumigatus.

Another uncommon feature of the presented case is the presence of thromboembolic disease. The patient suffered from DVT, recurrent pulmonary embolism, and subsequent right ventricular thrombus formation. It should be noted that thromboembolism is not usually recognized as a typical manifestation of Kartagener syndrome. In the presented case, a number of acquired factors contributing to the formation of a prothrombotic state can be suggested, among them chronic infection, systemic inflammation, hypoxemia, heart dysfunction, and subtherapeutic anticoagulation (INR 1.0). However, all these factors are nonspecific, and it is impossible to estimate the impact of each particular factor on the formation of recurrent thromboembolism based on a single case study.^[9,10]^

Positive U1-RNP antibody test adds one more potentially important, but still unconfirmed, feature to the case under discussion. It is known that U1-RNP antibodies indicate the presence of mixed connective tissue disease, especially when combined with the presence of typical clinical symptoms: Raynaud’s phenomenon, inflammatory arthritis, hand swelling, myositis, and sclerodactyly. However, none of the listed symptoms were found in the case study, and there was insufficient data to diagnose mixed connective tissue disease according to the classification criteria. Pericardial effusion, pulmonary hypertension/right ventricular dysfunction, and myocardial dysfunction can develop in the course of connective tissue disease, but are nonspecific and can also develop in cardiopulmonary disease. Thus, it can be stated that U1-RNP positivity is an indication of a probable autoimmune disorder but does not confirm its diagnosis in this case.^[11]^ Experimental and clinical studies have indicated a connection between U1-RNP-related immune mechanisms and endothelial dysfunction, but these observations do not confirm the role of this mechanism in the development of cardiovascular disorders in the case study.^[12]^

Thus, the following possible interacting diseases or processes can be suspected in this case: Kartagener syndrome and chronic bronchiectasis might have created a structural basis for Aspergillus-related pulmonary mycetoma; chronic infection, inflammation, hypoxemia, heart dysfunction, and subtherapeutic anticoagulation might have increased the risk of thromboembolism; and U1-RNP positivity might indicate a probable autoimmune disorder. These connections should be considered as hypotheses and not facts. It cannot be excluded that a coincidental combination of several independent diseases and other causes can be behind the clinical picture of the case.

## 4. Limitations

There are some issues with this case report. Firstly, there is no proof of Kartagener syndrome/ primary ciliary dyskinesia because the advanced tests, such as the measurement of nasal nitric oxide level, transmission electron microscopy of ciliary ultrastructure, and genetic testing, were unavailable at the institution when the disease was diagnosed. Secondly, despite the presence of U1-RNP antibodies, the clinical and serological data do not meet the established classification criteria for mixed connective tissue disease; thus, the autoimmune etiology of the condition cannot be proved. Thirdly, despite the negative results of the thrombophilia work-up, the role of chronic inflammation, hypoxemia, cardiac dysfunction, and suboptimal anticoagulation in causing the recurrent thromboembolic episodes cannot be assessed. Finally, although the patient’s condition improved during the six-month follow-up the patient’s condition improved, and the pulmonary cavity remained stable radiologically, it is necessary to conduct a follow-up in order to assess the structural outcome of the pulmonary mycetoma.

## 5. Conclusion

This is an example of the rare combination of Kartagener syndrome, pulmonary mycetoma in association with Aspergillus, recurrent thromboembolism, heart failure, and U1-RNP seropositivity. While chronic lung disease, infections, inflammation, hypoxia, inadequate anticoagulation, and potential autoimmune factors might have been responsible for the clinical picture, any causative link cannot be proven in one case. U1-RNP positive status suggests the presence of connective tissue disease overlap but does not imply a diagnosis of mixed connective tissue disease. The case underscores the significance of a multidisciplinary approach that includes microbiologic analysis of cavitary lung lesions, adequacy of therapeutic anticoagulation, thrombophilia workup, and autoimmunity testing as needed.

## Acknowledgments

Not applicable

## Author contributions

**Data curation:** Muhammad Asghar Khan, Okasha Tahir, Muhammad Hassaan Javaid, Eeman Shehzad, Tayyeb Ali.

**Formal analysis:** Muhammad Asghar Khan, Maheen Sheraz, Muhammad Hassaan Javaid, Laleen Altaf.

**Resources:** Muhammad Asghar Khan, Okasha Tahir, Hasibullah Aminpoor.

**Software:** Muhammad Asghar Khan, Abbas Khan, Maheen Sheraz, Eeman Shehzad.

**Writing – original draft:** Muhammad Asghar Khan, Muhammad Hassaan Javaid.

**Conceptualization:** Okasha Tahir, Muhammad Hassaan Javaid, Muhammad Ali, Tayyeb Ali.

**Project administration:** Okasha Tahir, Khalid Reza, Hasibullah Aminpoor.

**Supervision:** Abbas Khan, Maheen Sheraz, Khalid Reza.

**Validation:** Abbas Khan, Maheen Sheraz, Khalid Reza, Laleen Altaf.

**Visualization:** Abbas Khan, Khalid Reza, Laleen Altaf, Eeman Shehzad.

**Methodology:** Khalid Reza.

**Writing – review & editing:** Muhammad Hassaan Javaid.

**Investigation:** Laleen Altaf, Eeman Shehzad.

## References

[1] Poudel S Basnet A Bista S Shah R Chhetri BT. Kartagener’s syndrome with recurrent respiratory infection: a case report. Ann Med Surg (2012). 2023;85:3102–5. 10.1097/MS9.0000000000000796 PMC10289593 37363497

[2] Eitler K Bibok A Telkes G. Situs inversus totalis: a clinical review. Int J Gen Med. 2022;15:2437–49. 10.2147/IJGM.S295444 PMC8901252 35264880

[3] Nakagawa Y Shimazu K. A case of Kartagener’s syndrome associated with pulmonary tuberculosis, pneumothorax and pulmonary aspergilloma. Kansenshogaku Zasshi. 1998;72:553–6. 10.11150/kansenshogakuzasshi1970.72.553 9642947

[4] Ashitani J Mashimoto H Wakisaka A. Kartagener’s syndrome with aspergilloma. Nihon Kyobu Shikkan Gakkai Zasshi. 1997;35:371–4. 9168658

[5] Awasthi S Mishra S. Primary ciliary dyskinesia: a review. Rare. 2025;3:100098.

[6] Taha R Feteih M. Pulmonary Manifestations of Connective Tissue Diseases. In: Skills in Rheumatology. 2021:139–175. 36315804

[7] Bachh AA Haq I Gupta R Varudkar H Ram MB. Pulmonary candidiasis presenting as mycetoma. Lung India. 2008;25:165–7. 10.4103/0970-2113.45285 PMC3019347 21264087

[8] Kuek LE Lee RJ. First contact: the role of respiratory cilia in host-pathogen interactions in the airways. Am J Physiol Lung Cell Mol Physiol. 2020;319:L603–19. 10.1152/ajplung.00283.2020 PMC7516383 32783615

[9] Pati I Masiello F Piccinini V. Risk of venous thromboembolism in infectious diseases: a literature review. Pathogens. 2025;14:816. 10.3390/pathogens14080816 PMC12389338 40872326

[10] Paradowska-Gorycka A. U1-RNP and TLR receptors in the pathogenesis of mixed connective tissue disease: part I. Reumatologia. 2015;53:94–100. 10.5114/reum.2015.51509 PMC4847272 27407234

[11] Sapkota B Al Khalili Y. Mixed Connective Tissue Disease. In: StatPearls. StatPearls Publishing; 2024. 31194355

[12] Soltész P Bereczki D Szodoray P. Endothelial cell markers reflecting endothelial cell dysfunction in patients with mixed connective tissue disease. Arthritis Res Ther. 2010;12:R78. 10.1186/ar2999 PMC2911856 20459625

